# Optimizing agent-based transmission models for infectious diseases

**DOI:** 10.1186/s12859-015-0612-2

**Published:** 2015-06-02

**Authors:** Lander Willem, Sean Stijven, Engelbert Tijskens, Philippe Beutels, Niel Hens, Jan Broeckhove

**Affiliations:** Centre for Health Economics Research & Modeling of Infectious Diseases, Vaccine and Infectious Disease Institute, University of Antwerp, Antwerp, Belgium; Modeling of Systems And Internet Communication, Department of Mathematics and Computer Science, University of Antwerp, Antwerp, Belgium; Interuniversity Institute for Biostatistics and statistical Bioinformatics, Hasselt University, Hasselt, Belgium; Department of Information Technology, Ghent University–iMinds, Ghent, Belgium; HPC core facility CalcUA, Computational Mathematics, University of Antwerp, Antwerp, Belgium; School of Public Health and Community Medicine, The University of New South Wales, Sydney, Australia

**Keywords:** Mathematical epidemiology, Agent-based model, Optimization, Performance

## Abstract

**Background:**

Infectious disease modeling and computational power have evolved such that large-scale agent-based models (ABMs) have become feasible. However, the increasing hardware complexity requires adapted software designs to achieve the full potential of current high-performance workstations.

**Results:**

We have found large performance differences with a discrete-time ABM for close-contact disease transmission due to data locality. Sorting the population according to the social contact clusters reduced simulation time by a factor of two. Data locality and model performance can also be improved by storing person attributes separately instead of using person objects. Next, decreasing the number of operations by sorting people by health status before processing disease transmission has also a large impact on model performance. Depending of the clinical attack rate, target population and computer hardware, the introduction of the sort phase decreased the run time from 26 % up to more than 70 %. We have investigated the application of parallel programming techniques and found that the speedup is significant but it drops quickly with the number of cores. We observed that the effect of scheduling and workload chunk size is model specific and can make a large difference.

**Conclusions:**

Investment in performance optimization of ABM simulator code can lead to significant run time reductions. The key steps are straightforward: the data structure for the population and sorting people on health status before effecting disease propagation. We believe these conclusions to be valid for a wide range of infectious disease ABMs. We recommend that future studies evaluate the impact of data management, algorithmic procedures and parallelization on model performance.

**Electronic supplementary material:**

The online version of this article (doi:10.1186/s12859-015-0612-2) contains supplementary material, which is available to authorized users.

## Background

Agent-based models (ABMs) offer endless possibilities to explore heterogeneous problems and spatial patterns but come with a large computational burden. ABMs are increasingly used to model infectious disease transmission, but little attention is given in the literature to model implementation and performance, e.g., in [1-10]. Usually the simulation time on large clusters is mentioned, but it is not clear whether computational resources are optimally used. However, computational performance is a significant aspect of a simulators’ usefulness. Especially model exploration and sensitivity analysis, which require bulk calculations, benefit from efficient algorithms [11,12]. Furthermore, improving model performance facilitates model development and testing on workstation systems.

Performance is implementation specific and therefore we compared different close-contact infectious disease simulators starting from two published ABMs for pandemic influenza: FluTE from Chao *et al.* [6] and FRED (a Framework for Reconstructing Epidemic Dynamics) from Grefenstette *et al.* [10]. Both simulators are written in C++ and are free, open source software (FOSS) under the GNU General Public License and the BSD 3-Clause, respectively. The FluTE population model consists of census tracts with communities of 2000 residents on average. The simulation runs in discrete time steps of 12-h representing daytime with work, school and day community contacts and nighttime with household and home community contacts. All children go to school in the home community and adults are assigned to workplaces based on employment rates and commuting data. The community is the central unit in FluTE and one person is assigned to only one community per time step. The implementation of FRED is based on specific places for social contacts. Different places are used ranging from small households and classrooms to large schools and communities. All members of one place can have social contacts and one person might be assigned to multiple places per time step.

Individual behavior, social contact structures and population setup are very important to simulate infectious diseases. ABMs are suited to model these features because each person can be represented and stored separately. Inherent to these models are many checks and data transfers compared to the number of floating point calculations. For many years, hardware developers have been able to increase the central processing unit (CPU) performance [13]. Mass storage and memory subsystems have improved more slowly for cost reasons, which has introduced a performance gap between processing and accessing data. To reduce this imbalance, a hierarchy of small high-speed cache memories has been added to the CPU. Instead of fetching data multiple times from the main memory, it is loaded into cache and re-used [14]. The processor loads data into the cache in chunks called cache lines, which leads to efficient processing if in addition to one memory location also the nearby locations are referenced in the near future. This memory characteristic is important for the data layout of software [15]. For example, if person data is stored jointly in a person object (“Array of Structs") and next to a person’s age also their gender and zip-code are checked, it will already be available in the high-speed cache. On the other hand, if person attributes are stored in separate containers (“Struct of Arrays") and only the ages are checked, many more ages are available in one cache line and less slow memory accesses are required.

High-speed memory and other advances in CPU technology have enabled performance improvements for sequential software with about a factor of two for every eighteen months during a few decades [14]. Unfortunately, these improvements have now encountered physical limits and processor manufacturers have turned to multi-core and hyper-threading architectures to increase the accumulated peak performance [16]. These novel architectures require adaptations of existing software and new programming approaches to fully exploit the performance potential. Extra attention is needed for shared resources [17] like population data or random numbers.

Random numbers are a key resource of Monte Carlo methods and the more randomness they exhibit, the better [18]. Computer algorithms are by definition deterministic procedures. They can only approximate randomness by generating a stream of so-called pseudo-random numbers. The only true randomness in a sequence of pseudo-random numbers is the “seed” value that gets the series started. The complexity increases even more with parallel simulations. Some good pseudo-random number generators (PRNG) lose their efficiency or quality, or even both, when they are parallelized [19]. In parallel applications, independent streams of random numbers are required for each thread to prevent latency. Different parallelization techniques are used in practice. In “random seeding”, all processes use the same PRNG but with a different seed with the hope that they will generate non-overlapping series. More robust and versatile is the “leapfrog” method where one PRNG sequence is distributed (see Methods).

In this paper, we focus on single- and multi-core performance of discrete-time ABM simulators implemented in C/C++ to simulate infectious disease transmission. We used a limited close-contact disease simulator as case study. However, the features that we look into are also applicable to more extensive models or other types of ABMs. We investigate data management, algorithmic procedures and parallelization. We illustrate good-practice of a PRNG in a parallel context. The goal of this paper is to formulate recommendations for ABM simulators that are straightforward to realize and significantly benefit the performance.

The paper is structured as follows: First, we describe the methods starting with three different implementations of the population based on a general data structure. Second, we define an extension by adding a sorting algorithm. Third, we specify methods to run simulations in parallel with a shared-memory approach. Fourth, we describe the input data, run parameters and the work environment we used. Next, the Results and discussion section presents all findings. Finally, we end with concluding remarks and avenues for further research.

## Methods

### Model structure and implementation

**Fig. 1:**
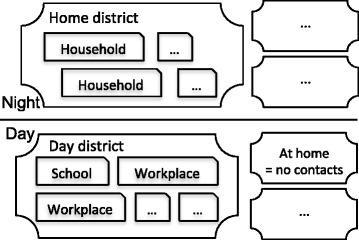
Social contact structure. People are member of a household cluster and the corresponding home district at night. During daytime, people can stay at home or go to a school or workplace in a day district.

We have opted for a model structure consisting of households, schools, workplaces and districts similar to published studies [6,10]. Figure 1 shows a schematic overview of the locations, which represent a group of people we define as a “cluster”. Social contacts can only be made within a cluster. During nighttime, people can have social contacts with members of their household and home district. During daytime, people stay at home or go to a workplace or school depending on their age, which also determines their day district. Contact between infectious and susceptible people may lead to disease transmission, which is a stochastic process based on social contact rates, infectiousness and susceptibility.

**Fig. 2:**
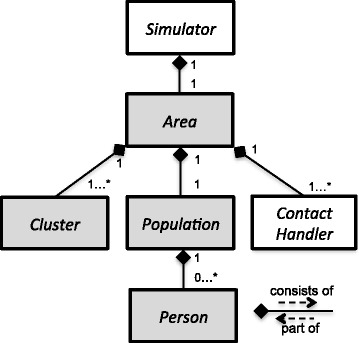
Model design: classes and compositions. The digits represent the number of links that are possible. E.g., the *Area* can have 1 or many (*) *Cluster* objects, but a *Cluster* can only be part of 1 *Area*. The models differ in the implementation of the grey classes: FLUTE has less *Cluster* types in *Area* and the *Population* in SID does not contain *Person* objects.

Figure 2 presents the model implementation with a general class diagram. We use a *Simulator* to organize the activities from the people in the *Area*. The *Area* has a *Population*, different *Cluster* objects and a *Contact Handler*. The *Contact Handler* performs Bernoulli trials based on the age of the contacts and random numbers. We included a 2 ×2 social contact matrix, based on literature [20-22], in which the transmission rate is doubled for contacts between children (<18 y). Each *Cluster* contains links to its members. The *Population* stores all person data (id, age, household, home district, day cluster, day district and health related parameters) within or without *Person* objects but we elaborate further on this issue in the next paragraph. An infection is assumed to follow a temporal pattern of Susceptible-Exposed-Infectious-Recovered (SEIR) states similar to an influenza-like disease [6,10]. After infection, people need 2 days of latency (infected but not infectious) before becoming infectious and 6 days to recover and acquire immunity against future infections.

We have constructed three implementations for the previous described transmission model: “FLUTE” and “FRED” are based on the corresponding open source models and “SID” has a novel data layout. The *Area* in FLUTE contains only home and day district *Clusters*. Membership to smaller sub-clusters like households, schools and workplaces can be retrieved from stored cluster IDs in *Person*. People in a district that are also member of the same sub-cluster have two opportunities for social contact and transmission. Therefore, during the processing of social contacts in the district, sub-cluster IDs need to be checked. If two people from a district are also member of the same sub-cluster, we used an aggregated transmission probability instead of performing two random draws. The *Area* of FRED and SID has also separate households and day *Clusters* (= workplaces and schools). We illustrate the difference with the following pseudo-code for the social contacts during nighttime with age dependent transmission probability P _*tr*_ and P _*tr*∗_ for one or two social contacts respectively:



### Population structure

Data for an individual is stored as a *Person* object in FLUTE and FRED and the *Population* is a container of *Person* entities, stored consecutively in memory. In SID, the *Population* has a different container for each person attribute and the data of one person is always located at the same index in each of those different containers. For example, to access the age of person *i* in FLUTE or FRED we use “population[i].age” while in SID we use “population.ages[i]”.

Population data have been extracted from the 2010 U.S. Synthetic Population Database (Version 1) from RTI International [23,24] for Brooklyn and Nassau County, New York. Every county or state from this database can be used to obtain individual age, household, school and workplace data. People of 16 to 18 years of age with a school and work ID in the original database were assigned to the school to guarantee that people were assigned to only one day cluster. To compare different model implementations, we needed an extra social contact layer (Fig. 1). We have created home districts by adding households, sorted on ID, until a number of 2000 people was reached. We assumed that household IDs are based on geographic proximity and the threshold was adopted from Chao *et al.* [6]. The day districts have been created analogously. The Nassau population consists of 1.31 million people in 448 519 households and 140 861 day clusters. Brooklyn has 2.46 million people and the cluster sizes range from one up to 62 962 people. More details on the study populations are listed in Table 1.

**Table 1:** **Population statistics. Legend: [min - max] and (median)**

Name	Nassau, New York	Brooklyn, New York
Ages	[0 – 94] years	[0 - 94] years
Day districts	386	630
Home districts	656	1231
Day clusters	140 861	183 451
Households	448 519	916 831
Population size	1 313 103	2 463 651
Household size	[1 - 18] (3)	[1 - 16] (2)
Day cluster size	[1 - 25 339] (1)	[1 - 62 962] (2)
Home district size	[1565 – 2009] (2002)	[660 - 2 013] (2002)
Day district size	[1071 - 26 458] (2021)	[1370 - 62 962] (2002)

The population data file determined the initial ordering of the person data in the *Population* object. We used seven different orderings for the same population details: the original sequence from the RTI database, a fully randomized order and population data sorted according to household, day cluster, and both household (first) and day cluster (second), and vice versa. To minimize the effect of random draws, we created five different files for each ordering with a random component.

### Algorithmic extension: sorting

The open source models [6,10] process disease transmission by looping over all members of a cluster and if a member is infectious, to match them with all susceptibles. To reduce the total number of operations, we introduced a modified algorithm in which the members of a cluster are first sorted according health status before the infectious members are matched with the susceptible members. A newly infected member is moved ahead of the first susceptible. The member list obtains the following structure: First, recovered and infected (exposed and infectious) members and second, susceptible members. The following pseudo-code shows the sort algorithm for FRED and SID (the algorithm for FLUTE is structured analogously).



### Parallelization: scheduling

The OpenMP API is often used for shared memory parallel programming in C/C++ [25]. In this programming model, subsets of a process are managed independently (=threads) and share a global address space of a single or multiple processors which they read and write asynchronously. For each cluster type (household, day district,...) in an area, a person is a member of only one cluster. Therefore, clusters are stored per type so that these containers can be processed in parallel without synchronization. Parallel processing within one cluster would lead to synchronization overhead. The workload distribution over the threads can be static or dynamic [25]. With static scheduling, a fixed number of tasks are assigned to each thread. In dynamic scheduling, the workload is distributed over the idle threads until all tasks are done. We have used workloads in chunks of one and ten clusters.

### Inputs and work environment

We used a 2 × 2 transmission matrix and assumed that the transmission probability (P _*tr*_) is doubled for contacts between children (<18y) [20-22]. Similar to the literature [6,10], we estimated the relationship between P _*tr*_ and the basic reproduction number R _0_ by counting the number of secondary cases of one infected in a complete susceptible population with seven P _*tr*_ values. Based on 4000 realizations with seven P _*tr*_, we approximated R _0_ by exp(5507*P _*tr*_*-0.1911)*. The total run time depends on the clinical attack rate (AR, total fraction of the population initially at risk that got infected) and for this reason, we performed we performed benchmarks for a range of R _0_ values (1.1, 1.25, 1.4, 1.8 and 3). Each simulation was performed for 100 days. To start the epidemic, we infected a random fraction of the population. After testing seeding rates of 1e ^−2^, 1e ^−3^, 1e ^−4^ and 1e ^−5^, we observed limited impact on the number of cases for these ranges and selected 1e ^−4^ as baseline setting.

We included the pseudo-random number generator (PRNG) from an open source software package called TRNG [19,26], a portable and highly optimized library of parallelizable generators. To prevent synchronization and latency, independent streams of random numbers are required for each thread. We used the robust and versatile “leapfrog” method where the PRNG sequence is distributed over *p* processes by calculating for draw *i* the i*(p-1)th number in the sequence. There are no recommendations to select PRNG seeds to obtain different stochastic results, except that those seeds have to be different. Therefore, the run index has been used to seed the PRNG.

An extended class diagram and the free open source code can be found in Additional file 1 and Additional file 2 respectively. Additional file 3 contains a user manual to make use of the project software. During development, we used the Google C++ Testing Framework [27] to perform detailed tests. These tests were applied in automated fashion with every change in the code base via a continuous integration server [28]. The Templatized C++ Command Line Parser library [29] was used to transfer configurations to the executable. The project-code is standard C++11 throughout, independent of external libraries and portable over all platforms that have a GNU compiler (version 4.8 or later) available.

Timings presented in this paper were obtained from benchmarks on a cluster with Intel®; Xeon®; E5-2680 v2 2.80 GHz CPU’s (release Q3’13) from the HPC core facility CalcUA at the University of Antwerp. We confirmed our results with benchmarks on quad-core Intel®; Xeon®; W5580 3.2 GHz (release Q1’09) CPU’s and AMD Opteron®; 6274 CPU’s. The GNU compiler (4.8) was used in release mode with compiler optimization “-O3”. Additional file 4 contains more info on the hardware and extra results. The open source tool PerfExpert [30] was used for profiling, as installed on the CalcUA cluster.

We performed additional benchmarks to explore the effect of cluster size, dynamic clusters and increased model complexity on model performance. Methods and results can be found in Additional file 5.

## Results and discussion

The number of infected people is the dominant factor in determining the computational workload and the required simulation time. Therefore, we needed to incorporate distinct epidemic curves in our benchmarks by using different R _0_ values. Small deviations in the AR were observed for each R _0_ as a result of different stochastic paths with and without the sort algorithm and given the different processing in FLUTE. To prevent stochastic fade-out, which is not appropriate for benchmarks, we used relatively high epidemic seeding rates to introduce new infected people in the population [12]. The benchmarks all report elapsed wall clock times as is appropriate for parallel programs. All results in this paper are based on mean timings from 10 runs with a different random number generator seed. With intervention strategies, we expect more stochastic fade-out and would require more realizations. Benchmarks are performed on idle computing nodes and results on other hardware can be found in Additional file 4.

**Fig. 3:**
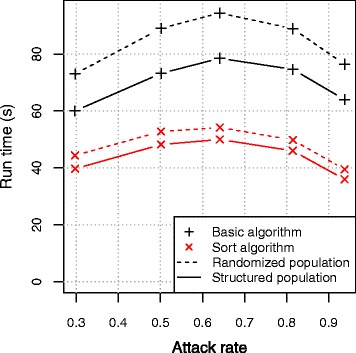
Run time according to attack rate and population structure for Nassau simulations using FRED. Structured population: sorted according to day cluster (first) and household (second).

Simulations with the basic models without concerns of the population order clearly required the longest run times. Figure 3 illustrates the total run time for FRED simulations with the Nassau population. Similar results were obtained with the other models (Additional file 4). We observed a large decrease in run time when the population is structured according to day cluster and household. The workload for a cluster of size *N* with *I* infectious and *S* susceptible members can be approximated by *N* health checks to select the infectious members + I*(N) health checks to select susceptible potential contacts + I*S random draws to match the infectious member with the susceptible members. The number of susceptibles decreases with each new case, which explains the decreasing curve in Fig. 3 for epidemics with high AR. Next, sorting cluster members on health status before processing disease transmission, had a large impact on the performance. The run times for Nassau were reduced with 26 % to 79 % compared to the basic models, depending on AR and population ordering. The algorithm with sorting has overhead because of swapping infected and healthy members but overcomes I*(N) health checks on susceptibility, which explains the reduced run times.

**Fig. 4:**
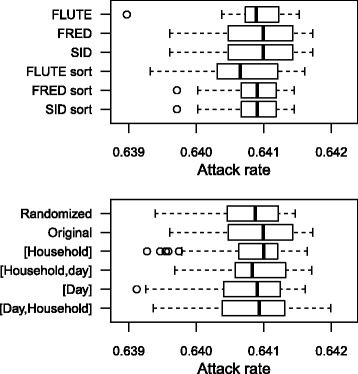
Attack rates for Nassau simulation using R _0_ = 1.4 and seeding rate = 1e ^−4^ according to implementation (top) and population structure (bottom). Results from 10 simulations. The original population structure is used to compare implementations (top) and FRED to compare population structures (bottom). Box: upper and lower quartile, wisker: minimum and maximum excluding outliers, circle: outliers (>1.5x interquartile range), […]: structured population e.g., [Day, Household] represents populations sorted according to day cluster (first) and household (second).

Given the impact of the AR on the simulation time, we needed to monitor the benchmarks closely. The stochastic transmission process is altered by the sorting algorithm, which has limited impact on the AR. Also, the population ordering determines the initial sequence of the cluster members and thus the random path of the simulator. Figure 4 presents the AR from 10 Nassau simulations using different models and population structures. The AR distributions were overlapping, which suggested similar transmission dynamics and approved run time comparisons. To validate the transmission model presented here, we performed simulations with the open source FRED software from Greffenstette et al. [10] using population data distributed with the source code for Allegheny, Pennsylvania. We observed ARs of ±68 % if R_0_=1.4 and ill people could not stay home, which was close to our results.

The population ordering appeared to have a large impact on model performance. To examine the effect on an epidemic with R _0_ = 1.4 (AR ±64 %), we used different versions of the population data with and without sorting according to household and/or day cluster. We repeated our benchmarks multiple times and did not observe large differences in ARs (Fig. 4). Table 2 presents the mean timings from multiple runs with each population ordering using the three basic models. The randomized populations gave the highest run times for all basic models. Using the original structure of the RTI population files slightly decreased the run time. Sorting the population on household ID improved the performance though most optimal was to sort the population on day cluster (first) and household (second). With this sorted population structure, we observed a reduction up to 59 % for FLUTE and FRED compared with the randomized population. The effect of the population structure was less for SID. The original open source FluTE model [6] uses a population sorted according to household. With our FLUTE implementation, we observed a decrease in run time of 20 % by using a population for Nassau sorted by day cluster and household. The population of the original FRED model [10] follows the structure of the RTI population files. A decrease of 6 % in total run time can be achieved with our FRED implementation by sorting the population file once. The impact of the population ordering was limited for the models with the sort algorithms.

**Table 2:** **Timings for Nassau simulations with different population structures**

Population structure	FLUTE	FRED	SID
Randomized	108	91	95
Original RTI sequence	101	94	92
[*Household*]	94	86	87
[Household, Day ]	93	80	87
[*Day*]	89	80	82
[Day, Household ]	81	69	78

Results in seconds with R _0_ = 1.4 and seeding rate = 1e ^−4^ (AR = ±62%). E.g., [Day, Household] represents populations sorted according to day cluster (first) and household (second)

The general trends from the Nassau simulations were also valid for Brooklyn. The improvement of the sorting algorithm ranged from 34 % to 63 %. For Brooklyn, we reduced the simulation time by sorting the population once with respectively 15 % and 19 % compared to the original FLUTE and FRED population. The highest improvement with the population structuring was 39 %. Table 3 presents the mean run times from 10 Brooklyn simulations with R _0_ = 1.4. The ranking of the basic models based on total run time differed between Nassau and Brooklyn simulations due to the different population size and cluster size distribution. For the models with the sorting algorithm in the cluster class, the ranking was independent of the population structure. The extra effort to manage separated household and day clusters in FRED and SID improved the simulators’ performance compared to the district-approach from FLUTE. The SID model with the sort algorithm performed best for all benchmarks, especially with the structured population.

**Table 3:** **Profiling results for Brooklyn simulations**

	FLUTE		FRED		SID	
	Basic	Sort	Basic	Sort	Basic	Sort
Randomized population						
- Branch instructions	0.29	0.14	0.23	0.07	0.18	0.07
- Data access	1.85	0.99	2.12	0.97	1.69	0.78
- LLC misses	0.14	0.14	0.27	0.48	0.13	0.29
- Run time (s)	229	114	237	103	222	102
[Day, Household ] population						
- Branch instructions	0.26	0.14	0.22	0.07	0.17	0.07
- Data access	1.23	0.85	1.42	0.67	0.66	0.35
- LLC misses	0.05	0.07	0.12	0.25	0.04	0.12
- Run time (s)	168	103	188	94	147	88

Results with R _0_ = 1.4 and seeding rate = 1e ^−4^ (AR = ±62%). All metrics, except run time, are given in LCPI: local cycles per instruction. LLC: last level cache. [Day, Household] populations are sorted according to day cluster (first) and household (second)

On today’s multi-core chips, memory access is a critical performance-limiting factor [31]. Therefore, we have analyzed software behavior and memory access patterns with a profiling tool for high-performance computing applications, PerfExpert [30]. We found that the function in *Cluster* to process disease transmission takes on average 98 % of the run time. Therefore, optimizations in this part of the code can have large impact. Since a member cannot be infectious and susceptible at the same time, it is not necessary to check whether a member tries to infect himself/herself. We observed that adding a simple comparison of two C++ pointers or two integer indices in FRED and SID respectively, increased the simulation time with 25 %. Table 3 presents a selection of the PerfExpert output. FLUTE had the highest penalty for branch instructions (if-then-else structures), which limits the CPU to pipeline instructions and to execute different stages (fetching, decoding, processing and store data) at the same time. A mispredicted branch instruction disturbs this optimization. FRED and SID required less cycles for branch instructions, especially with the sort algorithm. Sorting the cluster members before processing transmission also reduced the data access. Regarding the cache-coherency, we have observed that structuring the population according to the social contact clusters decreased the number of last level cache misses. The sorting algorithm disrupts the memory consistency by relocating references to cluster members. By comparing FRED and SID profiling results, we can confirm the targeted data management strategy from struct-of-array vs array-of-structs: the SID models have fewer last level cache misses.

**Fig. 5:**
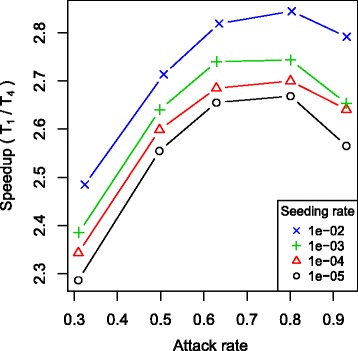
Speedup according to attack rate and epidemic seeding rate using FLUTE (basic) with 4 threads. All simulations were performed with a structured Brooklyn population sorted according to day cluster and household using dynamic scheduling with workload chunks of 1 cluster.

**Fig. 6:**
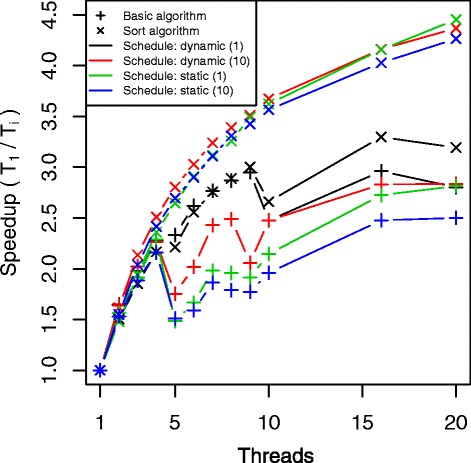
Speedup according to thread number and scheduling for Brooklyn simulations using SID. Results are shown for the basic and sort algorithm with dynamic and static parallel scheduling using workload chunk size of 1 and 10 clusters. All simulations were performed with a structured population sorted according to day cluster and household with R _0_ = 1.4 and seeding rate of 1e ^−2^ (AR = ±64 %).

Processing disease transmission requires many iterations over independent clusters and therefore seems suited for distributed programming. We observed that the effect of parallelization was dependent of the epidemic curve. Figure 5 presents differences in the speedup using FLUTE with 4 threads according to the AR and the epidemic seeding rate (= initial fraction of infected people). The different rates we used did not have impact on the total number of cases but only on the length of the initial phase with a small amount of infected clusters. Simulations with a high epidemic seeding rate and a large AR gave the best speedups using multiple threads. To illustrate the possibilities of parallelization, we compared simulation times using 1 to 20 threads for epidemics with R _0_ = 1.4 and seeding rate = 1e ^−2^ (AR ±64 %). Figure 6 presents the speedup for SID with basic and sort algorithm using a structured population according to day cluster and household. Similar results were obtained for the other implementations and using the randomized population, which can be found in Additional file 4. We observed good speedup for all models and scheduling options with 2 threads. With 3 or more threads, the added value of extra threads decreased due to memory bandwidth saturation. Making the clusters more self-contained by replacing the member references by actual person data would reduce this limitation although it requires much synchronization between the clusters and extra memory. All basic models had most benefit of dynamic scheduling with workload chunks of 1 cluster. With sorting, FRED and SID seemed to operate more optimally with static scheduling or dynamic scheduling with workload chunks larger than 1 cluster. For FLUTE, the dynamic scheduling with chunks of 1 cluster gave the best speedup. We tested the models on other hardware and observed similar results (Additional file 4).

By increasing model complexity, more different cluster types can be used and sorting the population might be less effective. If more person attributes are required for the disease transmission, co-locating these in a person object will be beneficial. On the other hand, the increased amount of person data will reduce the number of persons that fit in the high-speed cache, so more data needs to be fetched with higher latency. We explored these model aspects (Additional file 5) and observed that cluster size had a large impact on run time. Though, the differences regarding population sorting, model design (FLUTE, FRED and SID) and the sorting algorithm scaled with cluster size. To estimate the effect of dynamic clusters on model performance, we implemented a model with changing cluster membership over time. This way, the run time increased but the overall conclusions remained valid. Increasing model complexity by adding extra person attributes in FLUTE and FRED reduced the impact of the population sorting. The run times for SID remained constant if these attributes were not used, which confirmed the targeted data strategy of struct-of-array vs array-of-structs. The SID design became a disadvantage regarding model performance and workload for the programmer if these extra person attributes were involved in the transmission process.

## Conclusion

ABMs offer a very powerful and flexible framework to analyze infectious disease transmission. Unfortunately they come with a large computational cost. Investing time in code optimization and adaptation to hardware innovations reduces time available for adding new features although it can save much time during testing and in production.

We compared different ABM implementations for close-contact disease transmission models for two U.S. counties. Our ABM consisted of household, school, workplace and district clusters and people in a cluster can have social contacts and transmit an influenza-like disease. The transmission probability was assumed to be age dependent. We observed reductions up to 59 % by structuring the model population once according to the largest social contact cluster. Next, sorting the cluster members based on health status before processing disease transmission appeared also very beneficial for the model performance (reduction up to 79 % compared to the basic model).

Data movement and access require much more cycles than floating point operations and therefore data layout has impact on run time. We compared models that handle the population in large districts with models that also process the household and day clusters separately. The latter seemed beneficial for the performance especially in combination with the sorting on health status in the clusters. The storage of person data in separate containers instead of per person improved the data locality and cache-coherency and reduced modeling time. Models that sort cluster members on health-status before processing disease transmission are scalable with multiple threads if the epidemics have a limited initial phase. The parallel scheduling and workload chunk size had significant impact on the simulation time.

Increasing model complexity may reduce the impact of the population ordering. We describe the core of the simulator but more research is needed to assess the role of data layout and sorting algorithms together with mitigation strategies. Although improving data layout by using a separate container for each person feature might increase the model performance, it is counter intuitive for an ABM and requires extra effort from the modeler. The current software does not predict the workload before scheduling the chunks over multiple threads. We believe this scheduling would be a valuable extension to the parallel implementation because the cluster sizes and the amount of infected individuals per cluster can be very heterogeneous. In conclusion, large performance gains can be achieved with limited effort by structuring the population once, adding an algorithm that sorts by health status and selecting appropriate parallel settings.

## Additional files

Additional file 1
**Class diagram.** Schematic overview of the project.

Additional file 2
**Free open source code.** Documented C++ code with Makefiles.

Additional file 3
**User manual.** User manual of the project software.

Additional file 4
**Hardware specifications and extra results.** Computer hardware details and more benchmarks for Nassau and Brooklyn simulations.

Additional file 5
**Model exploration and validation.** Benchmarks to assess the impact of model complexity on model performance.

## Abbreviations

ABM: Agent-based model
AR: Attack rate
CPU: Central processing unit
PRNG: Pseudo-random number generator
