# Supplementary figures and images for: Optimizing agent-based transmission models for infectious diseases
Source: BMC Bioinformatics. 2015 Jun 2;16(1):183. doi: 10.1186/s12859-015-0612-2 (PMC4450454; doi:10.1186/s12859-015-0612-2)

Additional File 1 : Class diagram of the project

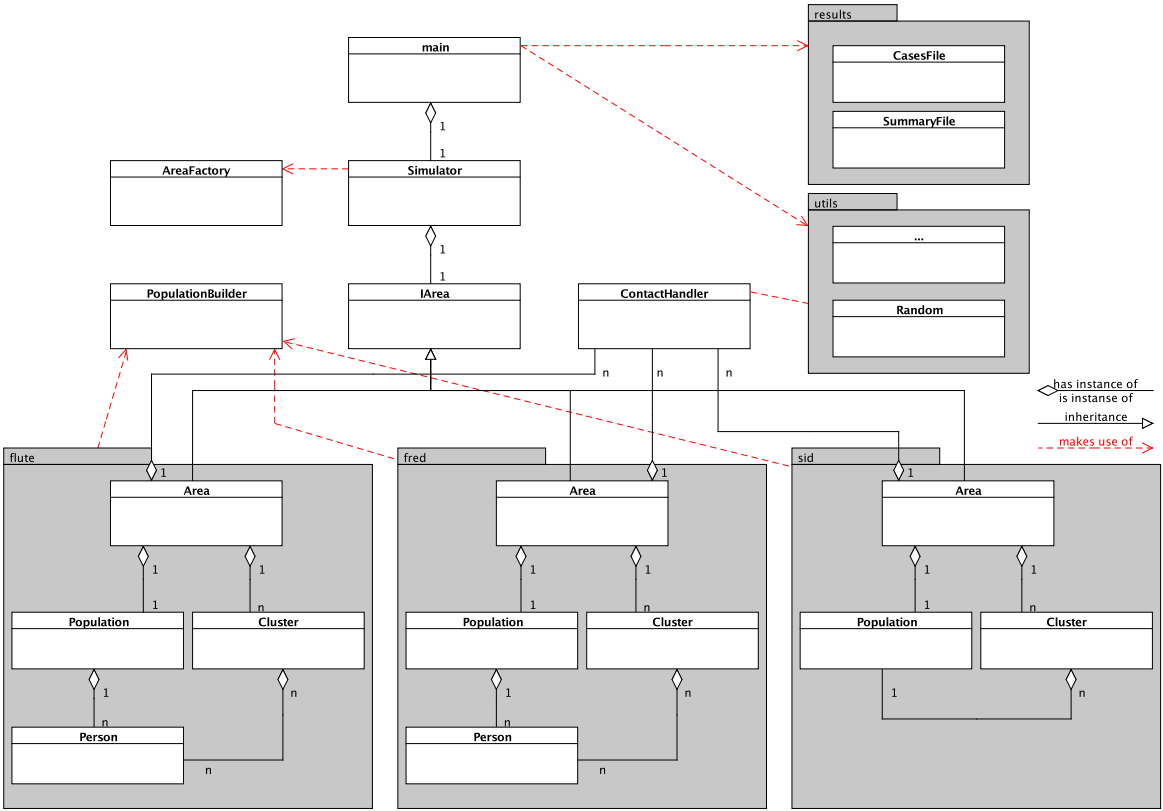

Supplement: Additional file 1 — Class diagram. Schematic overview of the project. [file 12859_2015_612_MOESM1_ESM.pdf]

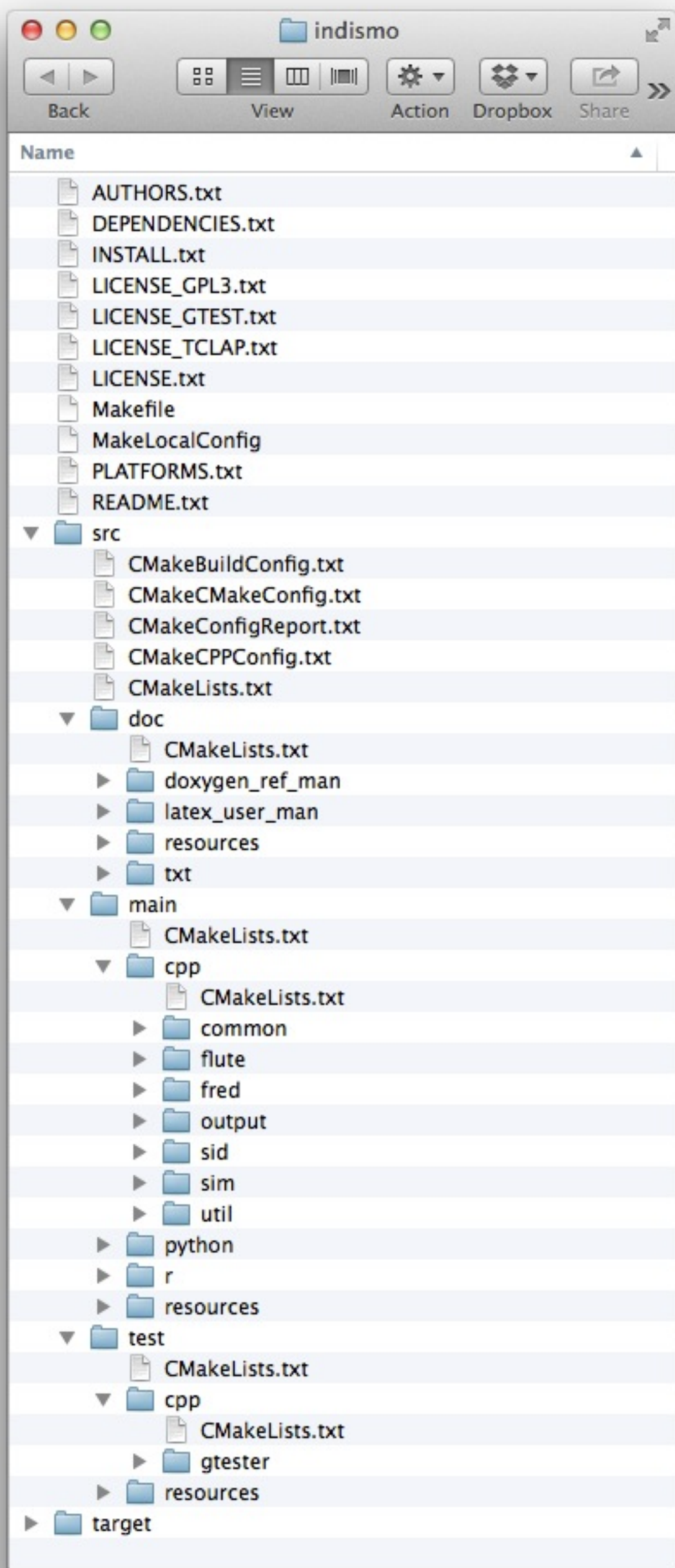

Supplement: Additional file 2 — Free open source code. Documented C++ code with Makefiles. [file 12859_2015_612_MOESM2_ESM.zip › indismo_software/src/doc/latex_user_man/images/screen_shot_main_dir.pdf]

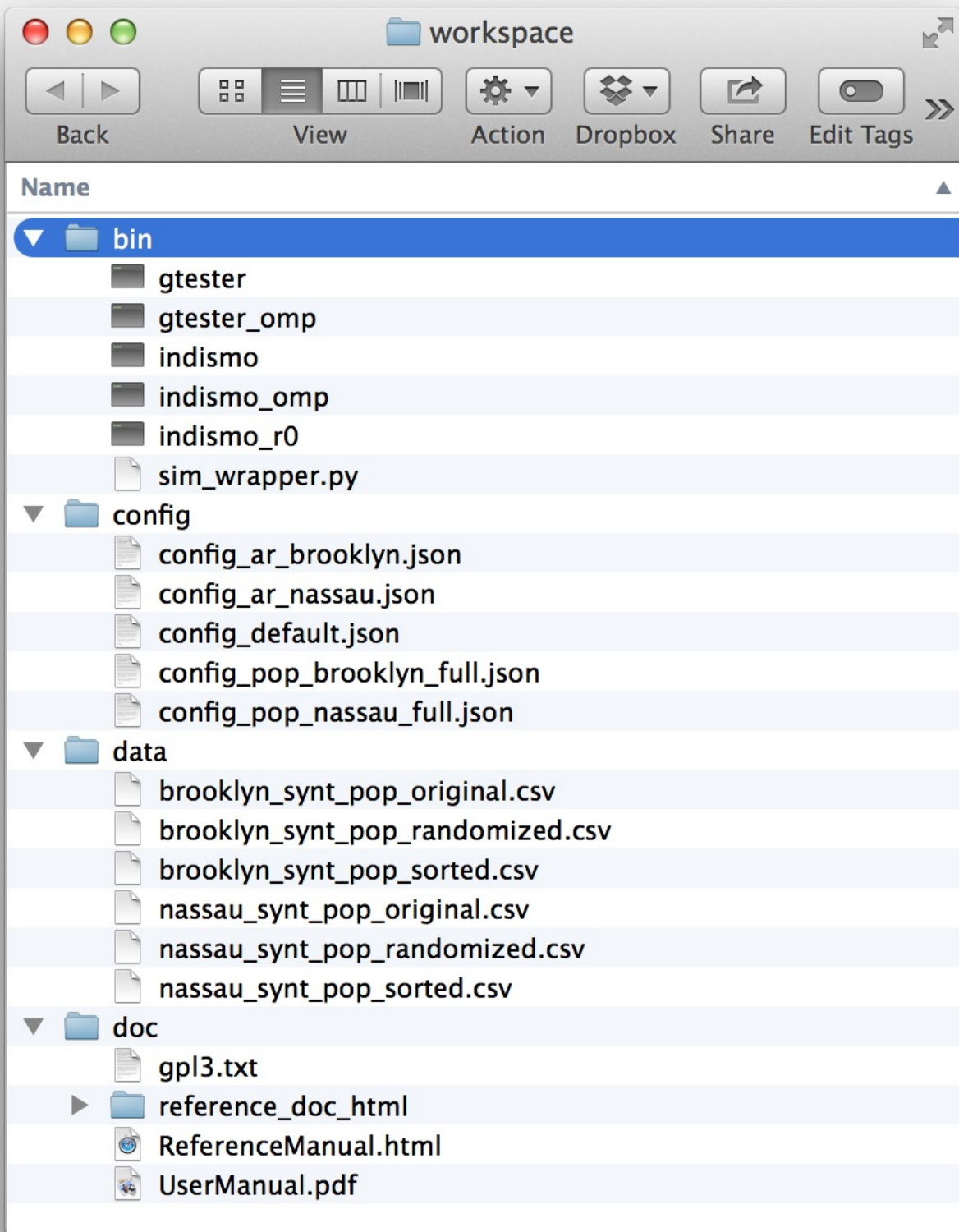

Supplement: Additional file 2 — Free open source code. Documented C++ code with Makefiles. [file 12859_2015_612_MOESM2_ESM.zip › indismo_software/src/doc/latex_user_man/images/screen_shot_workspace_dir.pdf]

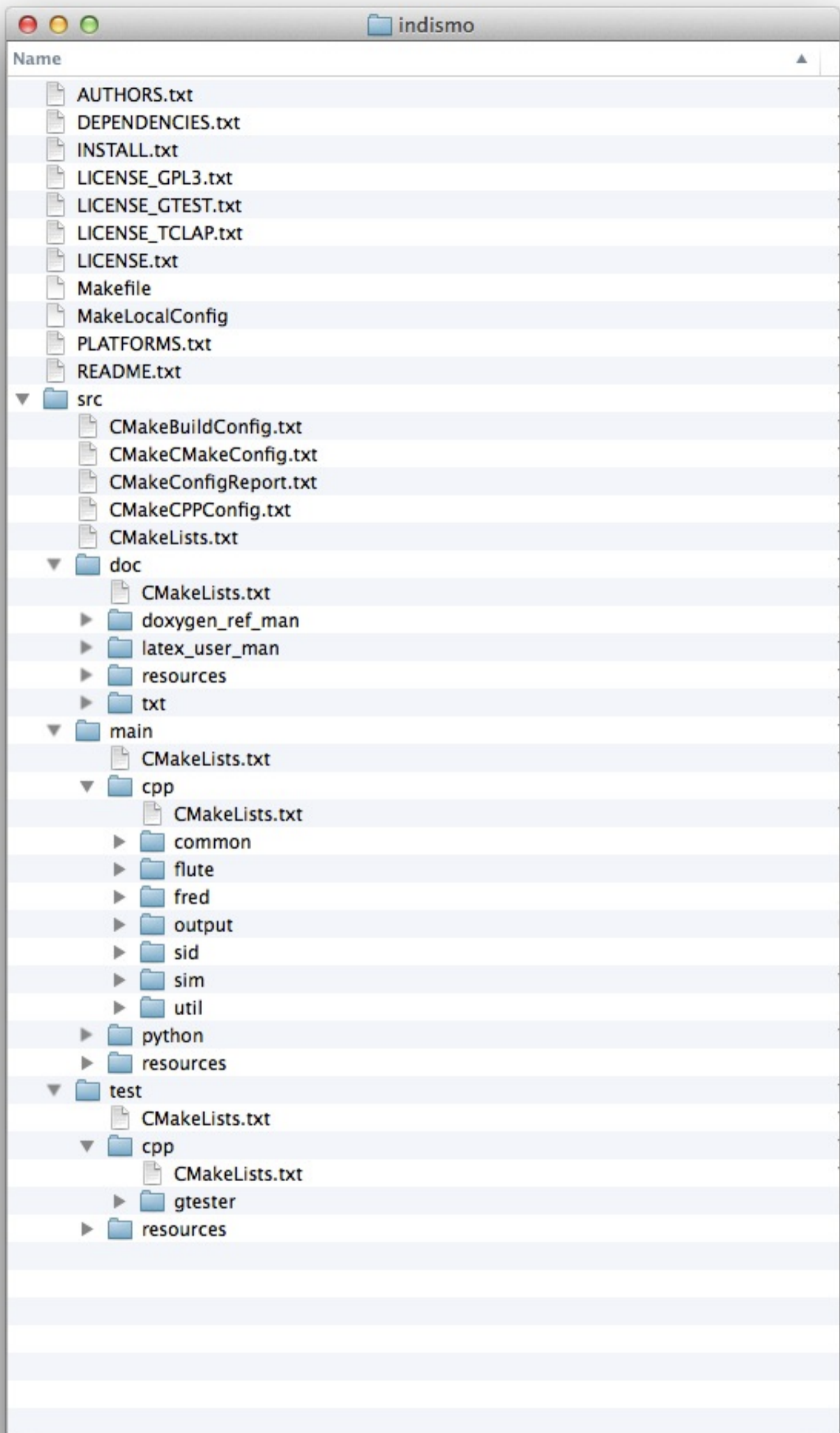

Supplement: Additional file 2 — Free open source code. Documented C++ code with Makefiles. [file 12859_2015_612_MOESM2_ESM.zip › indismo_software/src/doc/latex_user_man/images/screenshot_main_dir.pdf]
