# Supplementary material for: Optimizing agent-based transmission models for infectious diseases
Source: BMC Bioinformatics. 2015 Jun 2;16(1):183. doi: 10.1186/s12859-015-0612-2 (PMC4450454; doi:10.1186/s12859-015-0612-2)
Supplement: Additional file 2 — Free open source code. Documented C++ code with Makefiles. [file 12859_2015_612_MOESM2_ESM.zip › indismo_software/src/doc/doxygen_ref_man/ReferenceManual.html]

Page Redirection


If you are not redirected automatically, follow the link
