# Supplementary material for: Optimizing agent-based transmission models for infectious diseases
Source: BMC Bioinformatics. 2015 Jun 2;16(1):183. doi: 10.1186/s12859-015-0612-2 (PMC4450454; doi:10.1186/s12859-015-0612-2)
Supplement: Additional file 2 — Free open source code. Documented C++ code with Makefiles. [file 12859_2015_612_MOESM2_ESM.zip › indismo_software/src/doc/latex_user_man/images/screen_shot_config_file.pdf]

config_ar_nassau.json


```json
{
        "indismo_path":"bin/indismo",
        "rng_seeds":[1,2,3,4,5,6,7,8,9,10],
        "r0":[1.1, 1.25, 1.4, 1.8, 3],
        "seeding_rates":[0.0001],
        "population_files":["./data/nassau_synt_pop_sorted.csv"],
        "threads":[1],
        "days":100,
        "output":"ar_nassau",
        "omp_schedule":"DYNAMIC,1",
        "model":["all"]
}
```
