# Supplementary material for: Optimizing agent-based transmission models for infectious diseases
Source: BMC Bioinformatics. 2015 Jun 2;16(1):183. doi: 10.1186/s12859-015-0612-2 (PMC4450454; doi:10.1186/s12859-015-0612-2)
Supplement: Additional file 2 — Free open source code. Documented C++ code with Makefiles. [file 12859_2015_612_MOESM2_ESM.zip › indismo_software/src/doc/latex_user_man/images/screen_shot_doxygen_API.pdf]

Indismo Reference Manual: indismo

file:///Users/lwillem/opt/indismo2_v4-161+/doc/reference_doc_html/index.htm

Reader

Indismo Reference Manual

▼ Indismo Reference Manual
- ▶ indismo
- ▶ Namespaces
- ▶ Classes
- ▶ Files

indismo

Documentation

The web pages you are currently reading constitute the documentation for the current version of the indismo project.

- **Developer** information is generated from the source using the doxygen tool.
- **Reference** documentation also available in these pages.
- **Installation** information is provided in a text file in the distribution.
- **User** manual generated from latex sources and available in pdf format.

License

Indismo is free software: you can redistribute it and/or modify it under the terms of the GNU General Public License as published by the Free Software Foundation, either version 3 of the License, or (at your option) any later version. Indismo2 is distributed in the hope that it will be useful, but without any warranty, without even the implied warranty of merchantability or fitness for a particular purpose. See the GNU General Public License for more details.

A copy of the GNU General Public License can be found in the indismo project directories (file gpl3.txt) in a directory named doc. A copy of the license text is also available at the GNU web site, where you will also find background information on GNU software licenses.
