# Supplementary material for: Optimizing agent-based transmission models for infectious diseases
Source: BMC Bioinformatics. 2015 Jun 2;16(1):183. doi: 10.1186/s12859-015-0612-2 (PMC4450454; doi:10.1186/s12859-015-0612-2)
Supplement: Additional file 3 — User manual. User manual of the project software. [file 12859_2015_612_MOESM3_ESM.pdf]

Indismo project

User Manual

Version 1.0 (February 13, 2015) .

Centre for Health Economics Research & Modeling of Infectious Diseases, Vaccine and Infectious Disease Institute, University of Antwerp.

Modeling of Systems and Internet Communication, Department of Mathematics and Computer Science, University of Antwerp.

Interuniversity Institute for Biostatistics and statistical Bioinformatics, Hasselt University.

L.Willem, S.Stijven, & J.Broeckhove

Contents

1 Introduction **2**

2 Software **4**

2.1 System Requirements 4
2.2 Installation 4
2.3 Documentation 5
2.4 Directory layout 6
2.5 File formats 7
2.6 Testing 7
2.7 Results 7
2.8 Sim Wrapper 7

3 Simulator **8**

3.1 Workspace 8
3.2 Run the simulator 10
3.3 Sim Wrapper 10

CHAPTER 1

Introduction

This manual provides a brief description of the indismo software and its features. Indismo is an open source individual-based modeling system for close-contact disease transmission developed by researchers at the University of Antwerp and Hasselt University, Belgium. The simulator uses census-based synthetic populations that capture the demographic and geographic distribution, as well as detailed social networks. The software provides data structures and algorithms to model disease spreading in synthetic populations to compare and discuss model performance. Indismo is open source in the hope of making large-scale individual-based epidemic models more useful to the community. More info on the project and results obtained with the software can be found in the paper: “Optimizing Stochastic Individual-Based Models for Infectious Diseases” from Willem *et al* (2014).

The model population consists of households, schools, workplaces and districts, which represent a group of people we define as a “cluster”. Social contacts can only happen within a cluster. At night, people are present in their household and home district and can make social contacts with the other members. During daytime, people can be assigned to a workplace or school in a specific district or stay at home.

We use a Simulator class to organize the activities from the people in an Area. The Area class has a Population, different Cluster objects and a Contact Handler. The Contact Handler performs Bernoulli trials to decide whether a contact between an infectious and susceptible person leads to disease transmission. People transit through Susceptible-Exposed-Infected-Recovered states, similar to an influenza-like disease. Each Cluster contains a link to its members and the Population stores all person data.

The indismo software contains three core implementations of individual-based simulators:

FLUTE
: Implementation based on the open source model from Chao et al. [**?**]. The Area contains only clusters at the district level. The household and workplace or school ID needs to be checked whether two district members make extra contact. The Population is a collection of Person objects.

FRED
: Implementation based on the open source model from Grefenstette et al. [**?**]. The household, workplace and school clusters are handled separately from the district clusters, which are only used to model general community contacts. The Population is equally structured as in FLUTE.

SID
: Similar cluster structure as FRED but with an innovative data-layout in Population. The Population does not uses Person objects, but has a separate container for each person feature.

Next to the above described core differences, two algorithmic approaches are incorporated into the software to handle social contacts: with and without sorting the cluster members on health state. In total the indismo software enables the comparison of six different implementations.

CHAPTER 2

Software

2.1 System Requirements

Indismo is written in C++ and portable over all platforms that have the GNU C++ compiler. De software has no dependencies on external libraries. The following tools needs to be installed:

- g++
- make
- CMake
- Python (optional)
- Doxygen (optional, for documentation)
- LaTeX (optional, for documentation)

2.2 Installation

To install the project, first obtain the source code by cloning the repository to a directory (e.g. "git clone https://bitbucket.org/indismo/indismo") or download a zip file with all project material from the Bitbucket website and de-compress the archive. <<released after acceptance of the paper>> The build system for indismo uses the CMake tool. This is used to build and install the software at a high level of abstraction and almost platform independent (see `http://www.cmake.org/`). The project includes the conventional make targets to "build", "install", "test" and "clean" the project. There is one additional target "configure" to set up the CMake/make structure that will actually do all the work. For those users that do not have a working knowledge of CMake, a front end Makefile has been

provided that invokes the appropriate CMake commands. More details on building the software can be found in "INSTALL.txt" in the source folder.

2.3 Documentation

The Application Programmer Interface (API) documentation is generated automatically using the Doxygen tool from documentation instructions embedded in the code (see www.doxygen.org). The developer documentation or reference is written in Doxygen syntax and also generated to html format. Figure 2.1 presents the starting page of the API documentation. The user manual has been written in LaTeX(see www.latex-project.org) and is generated to PDF format.

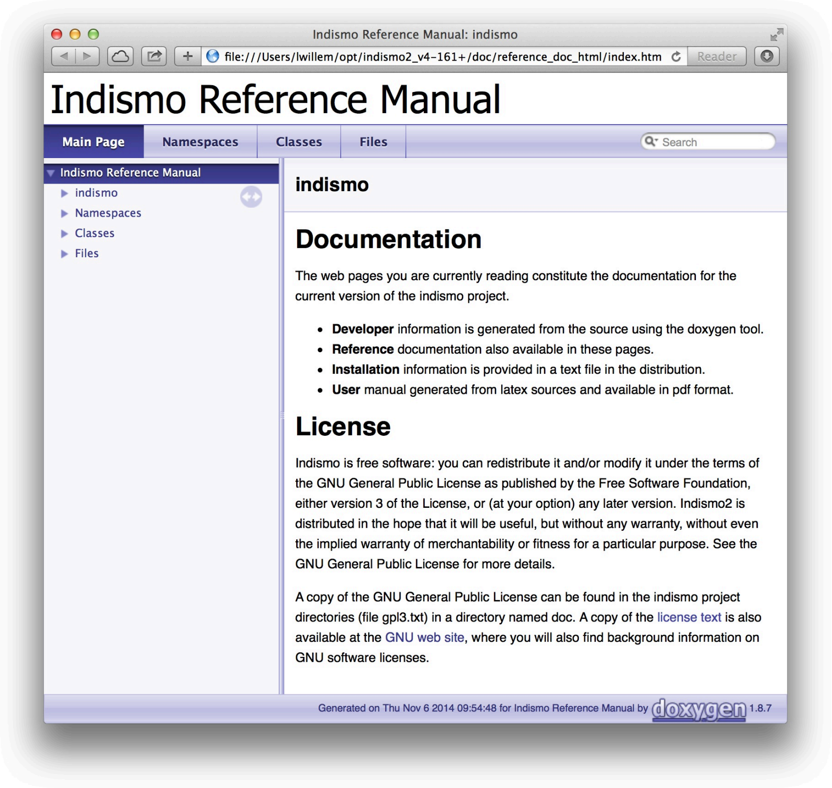

Figure 2.1: Screenshot of the main page of the API documentation generated with the Doxygen tool. The menu on the left provides access to documentation for individual classes.

2.4 Directory layout

The project directory structure has been designed systematically with a layout following maven conventions. The directory layout is represented in Figure 2.2

Everything used to generate project artefacts is placed in directory `src`:

- code related files (sources, third party libraries and headers, ...) in directory `src/main`
  - for each language the sources in `src/main/"language"...`
  - third party resources in `src/main/resources`.
- documentation files (api, manual, html, pdf and text ...) in directory `src/doc`
  - for each document processing tool a sub directory `src/doc/"tool"...`
- test related files (scripts, regression files, ...) in directory `src/test`

Every artefact is generated in directory `target` or its sub directories during the build procedure and is completely removed when the project is cleaned.

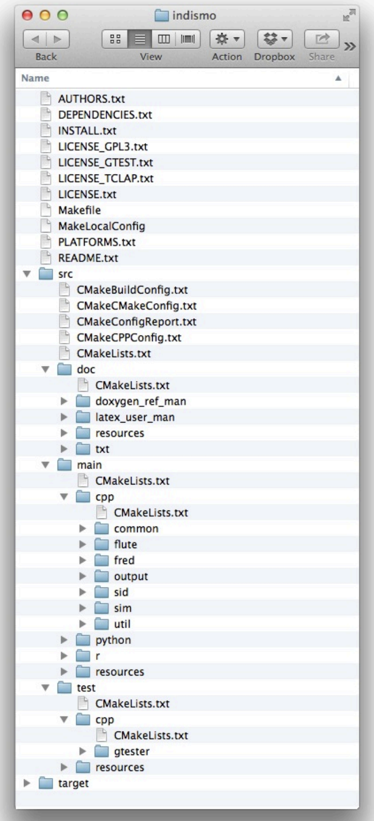

Figure 2.2: Screen shot of the main directory with the indismo source code.

2.5 File formats

The indismo software supports two file formats:

CSV or comma separated values, used for population input data and the simulator output.

JSON or JavaScript Object Notation, an open standard format that uses human-readable text to transmit objects consisting of attribute-value pairs.
(see `www.json.org`)

2.6 Testing

Using Google's `gtest` framework and CMake's `ctest` tool, unit tests and install checks have been added to indismo. In addition, the code base contains assertions to verify the simulator logic. They are activated when the application is built and executed in debug mode and are helpful in catching errors at run time.

2.7 Results

The software generates two files:

Log contains the cumulative number of cases per day.

Output contains aggregated results on the number of cases, configuration details and timings.

2.8 Sim Wrapper

The different model implementations have been captured into a single binary. This makes it possible to build wrappers in Python. A wrapper object can forward configurations to the C++ binary and merges the resulting output.

CHAPTER 3

Simulator

3.1 Workspace

By default, indismo is installed in `target/installed/` inside de project directory though this can be modified using the CMakeLocalConfig.txt file (example is given in `src/main/resources/make`). Compilation and installation of the software will create the following files and directories: (illustrated in Figure 3.1):

- Binaries in directory `bin`
  - gtester: regression tests for the sequential code.
  - gtester_omp: regression tests for the OpenMP code.
  - indismo: sequential executable.
  - indismo_omp: OpenMP executable.
  - indismo_r0: executable to measure the basic reproduction number (R_0).
  - sim_wrapper.py: the Python simulation wrapper (see section 2.8)
- Configuration files (json) in directory `config`
  - config_ar_brooklyn.json: configuration file for the sim_wrapper to perform Brooklyn simulations with different clinical attack rates.
  - config_ar_nassau.json: configuration file for the sim_wrapper to perform Nassau simulations with different clinical attack rates.
  - config_ar_nassau.json: default configuration file for the sim_wrapper with basic settings.
  - config_pop_brooklyn.json: configuration file for the sim_wrapper to perform Brooklyn simulations using population files with a randomized, sorted and original RTI person sequence.
  - config_pop_nassau.json: configuration file for the sim_wrapper to perform Brooklyn simulations using population files with a randomized, sorted and original RTI person sequence.

- Input data files (csv) in directory `data`
  - brooklyn_synt_pop_original: Synthetic population data extracted from The 2010 U.S. Synthetic Population Database (Version 1) from RTI International for Brooklyn, New York [? ?]. The original person ordering is retained.
  - brooklyn_synt_pop_randomized: Synthetic population data for Brooklyn in a randomized order.
  - brooklyn_synt_pop_sorted: Synthetic population data for Brooklyn sorted according to day cluster (first) and household (second).
  - nassay_synt_pop_original: Synthetic population data extracted from The 2010 U.S. Synthetic Population Database (Version 1) from RTI International for Nassau, New York [? ?]. The original person ordering is retained.
  - brooklyn_synt_pop_randomized: Synthetic population data for Nassau in a randomized order.
  - brooklyn_synt_pop_sorted: Synthetic population data for Nassau sorted according to day cluster (first) and household (second).
- Documentation files (api, manual, html, pdf and text ...) in directory `doc`
  - Refrence manual (see section 2.3)
  - User manual

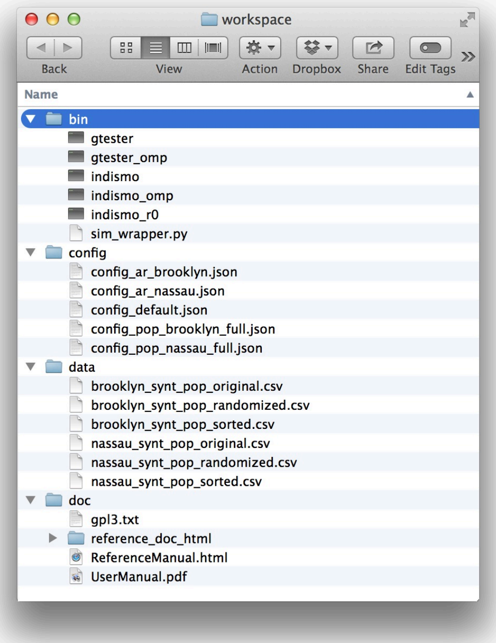

Figure 3.1: Screen shot of the workspace directory.

3.2 Run the simulator

From the workspace directory, the simulator can be started with default configuration using the command "`./bin/indismo`". Settings can be passed to the simulator using one or more command line arguments:

-m or --model Model you want to run: "flute", "flute_sort", "fred", "fred_sort", "sid", "sid_sort"

-o or --output_prefix Prefix for the output files, by default a time stamp.

-p or --population_file Population file.

-r or --r0 Basic reproduction number: the number of secondary cases by a typical primary case in a complete susceptible population.

-n or --rng_seed Random number generator seed.

-s or --seeding_rate Epidemic seeding rate: fraction of initially infected people to start the epidemic.

-t or --transmission_rate Transmission Rate: the probability that an infection is transmitted during a contact between two adults (+18 years) in the same social contact cluster.

-d or –days Number of days to simulate.

3.3 Sim Wrapper

A Python wrapper is provided to perform multiple runs with the C++ executable. The wrapper forwards the model configurations with command line arguments and merges the output. The wrapper is designed to be used with .json configuration files and examples are provided with the source code. E.g
"`./bin/sim_wrapper -- config ./config/config_ar_nassau.json`"
will start the simulator with each configuration in the file illustrated in Figure 3.2. It is important to note the input notation: values given inside brackets can be extended (e.g. "rng_seeds"=[1,2,3]) but single values can only be replaced by one other value (e.g. "days": 100).

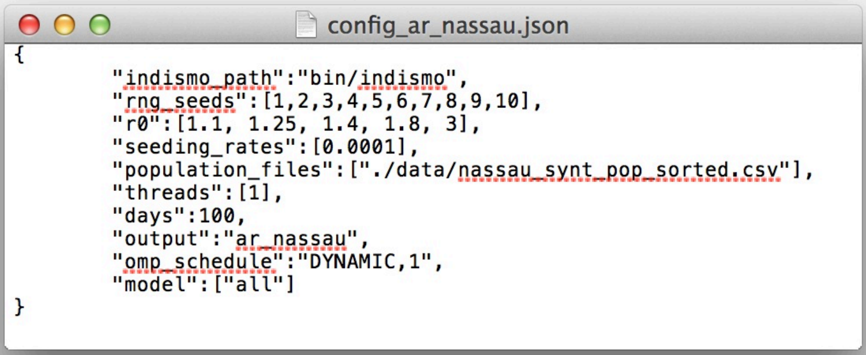
config_ar_nassau.json

```
{
	"indismo_path":"bin/indismo",
	"rng_seeds":[1,2,3,4,5,6,7,8,9,10],
	"r0":[1.1, 1.25, 1.4, 1.8, 3],
	"seeding_rates":[0.0001],
	"population_files":["./data/nassau_synt_pop_sorted.csv"],
	"threads":[1],
	"days":100,
	"output":"ar_nassau",
	"omp_schedule":"DYNAMIC,1",
	"model":["all"]
}
```

Figure 3.2: Screen shot of a json configuration file for the Python sim_wrapper.
