# Supplementary material for: Optimizing agent-based transmission models for infectious diseases
Source: BMC Bioinformatics. 2015 Jun 2;16(1):183. doi: 10.1186/s12859-015-0612-2 (PMC4450454; doi:10.1186/s12859-015-0612-2)
Supplement: Additional file 4 — Hardware specifications and extra results. Computer hardware details and more benchmarks for Nassau and Brooklyn simulations. [file 12859_2015_612_MOESM4_ESM.pdf]

Additional file 4 : Hardware specifications and extra results.

This additional file presents the hardware specifications of the used platforms and extra results in addition to the figures in the text. Figure S1 presents all benchmarks with the sequential models for the Nassau and Brooklyn population on CPU type 1. Figure S2 and S3 present similar results for CPU type 2 and 3 respectively. Figure S4 shows the run time according to the number of threads on CPU type 1 for all models with the brooklyn population. Figure S5 presents the OpenMP results for CPU type 2.

Hardware specifications

**Name**	**CPU type 1**	**CPU type 2**	**CPU type 3**
Configuration	Cluster	Desktop	Cluster
CPU type	Intel® Xeon® CPU E5-2680V2	Intel® Xeon® W5580	AMD Opteron® 6274
CPU speed	2.8 GHz	3.20 GHz	2.2 GHz
CPU cores	20 (2x10)	4	64 (4x16)
RAM	64 GB	24 GB	192 GB
L1 cache	10x64 KB	4x64 KB	16x48 KB
L2 cache	10x256 KB	4x256 KB	16x1024 KB
L3 cache	25 MB	8 MB	16 MB
Compiler	GNU gcc 4.8.2	GNU gcc 4.8.1	GNU gcc 4.8.1
Platform	Red Hat 4.4.7-3	Ubuntu 12.04.4 LTS	Ubuntu 12.04.1 LTS

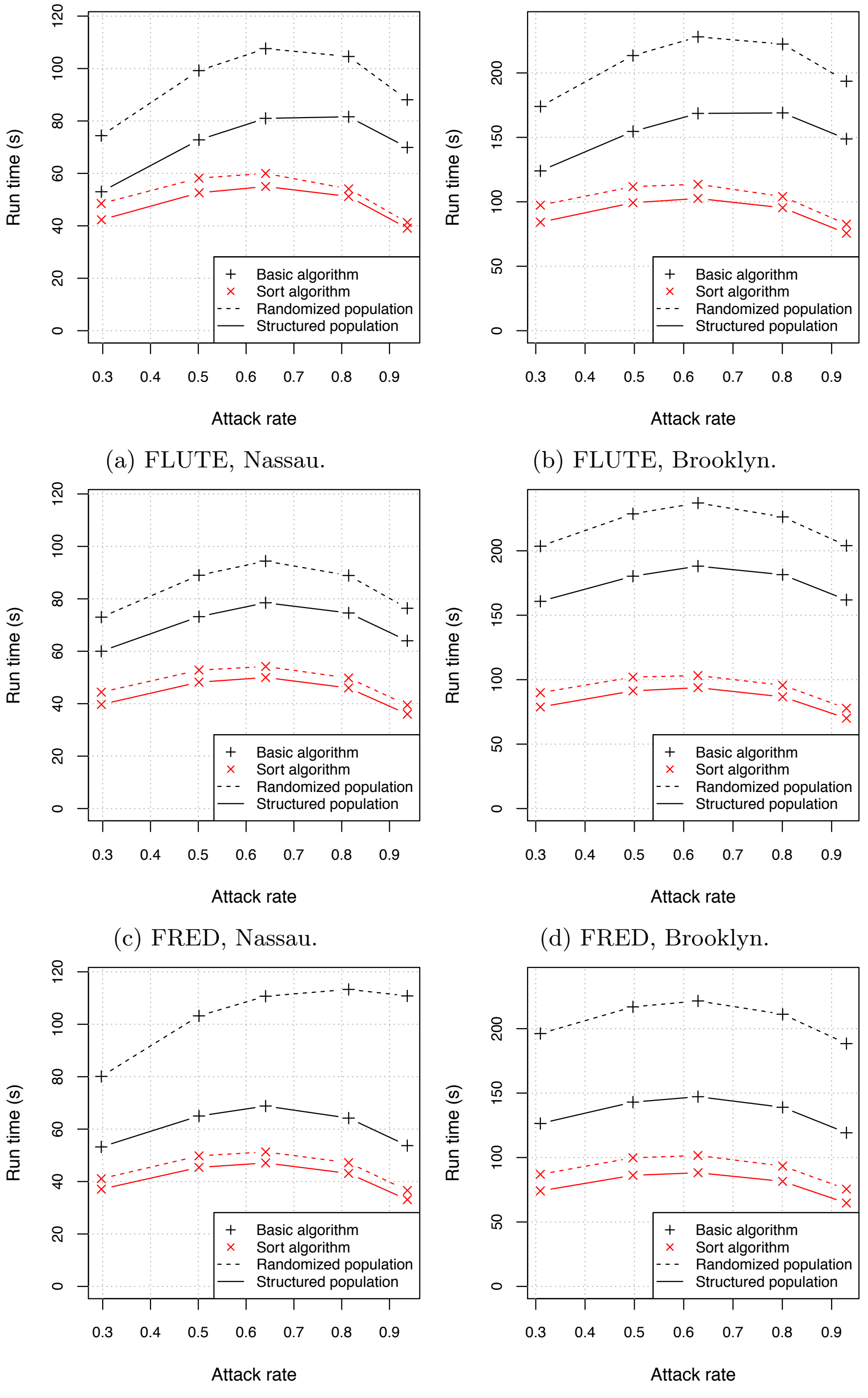

(a) FLUTE, Nassau.
(b) FLUTE, Brooklyn.
(c) FRED, Nassau.
(d) FRED, Brooklyn.
(e) SID, Nassau.
(f) SID, Brooklyn.

Figure S1: **Run time according to attack rate for Nassau and Brooklyn simulations on CPU type 1.**

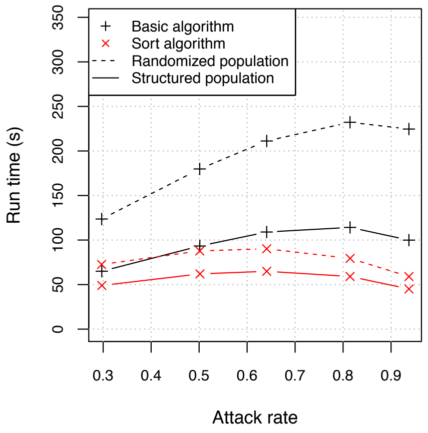

(a) FLUTE, Nassau.

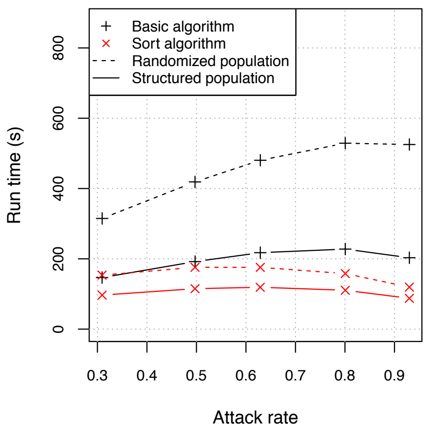

(b) FLUTE, Brooklyn.

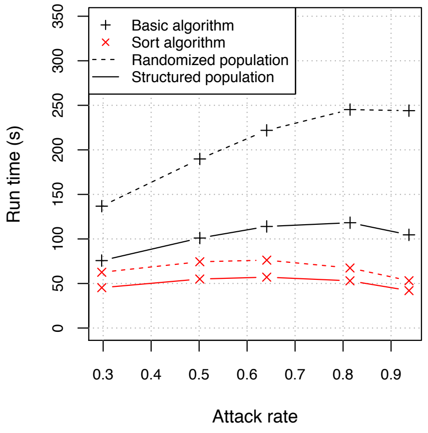

(c) FRED, Nassau.

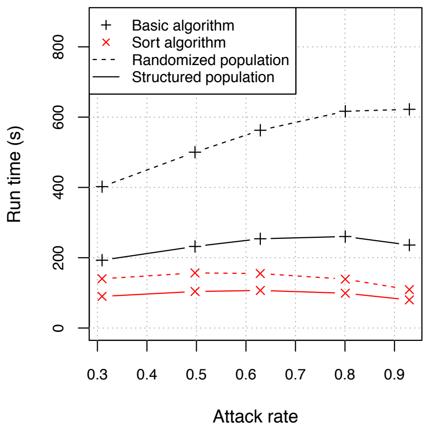

(d) FRED, Brooklyn.

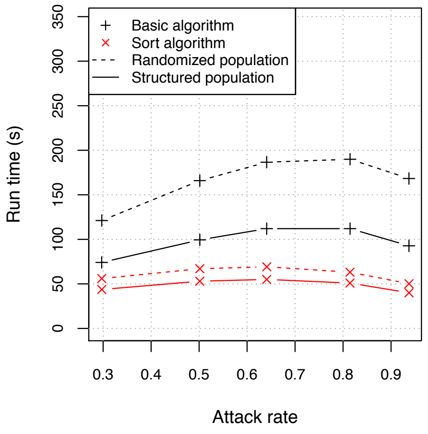

(e) SID, Nassau.

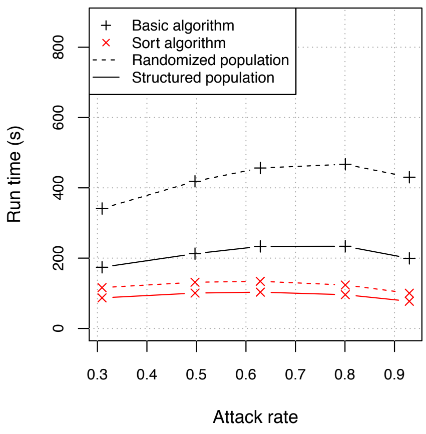

(f) SID, Brooklyn.

Figure S2: **Run time according to attack rate for Nassau and Brooklyn simulations on CPU type 2.**

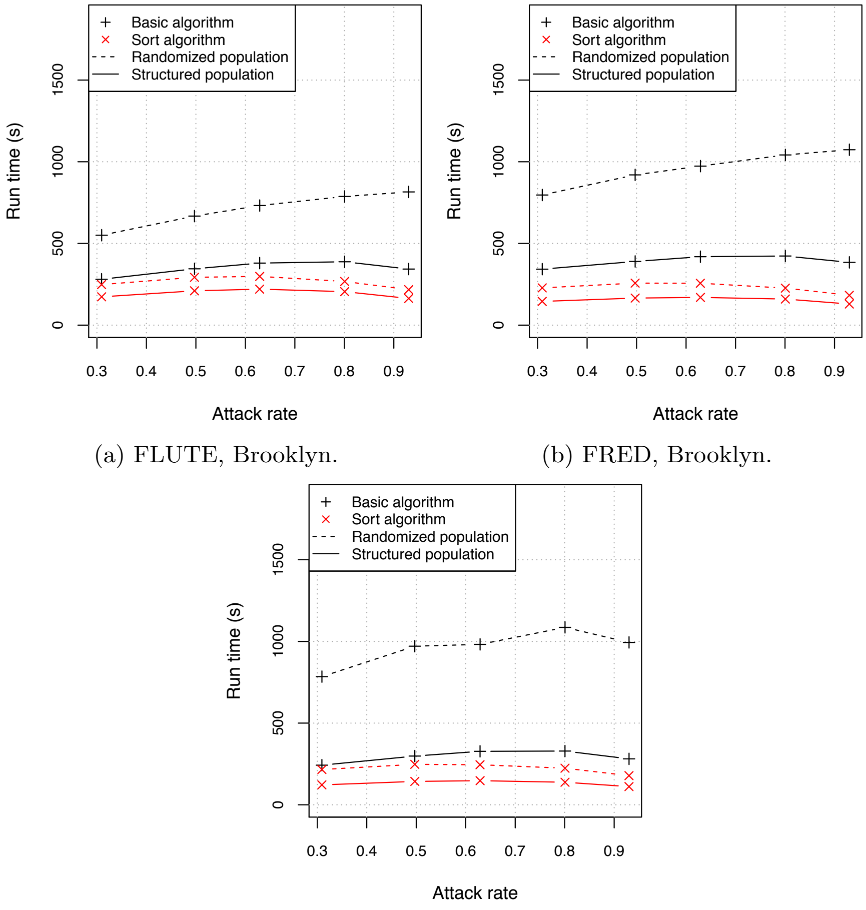

(a) FLUTE, Brooklyn.

(b) FRED, Brooklyn.

(c) SID, Brooklyn.

Figure S3: **Run time according to attack rate for Nassau and Brooklyn simulations on CPU type 3.**

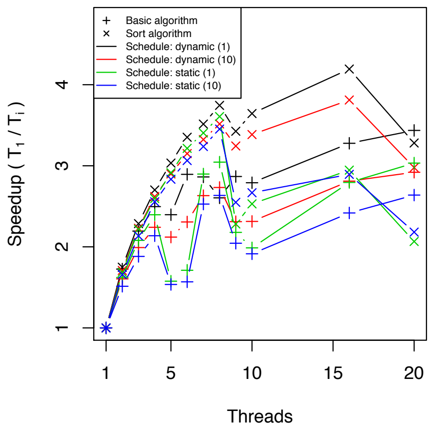

(a) FLUTE, sorted population.

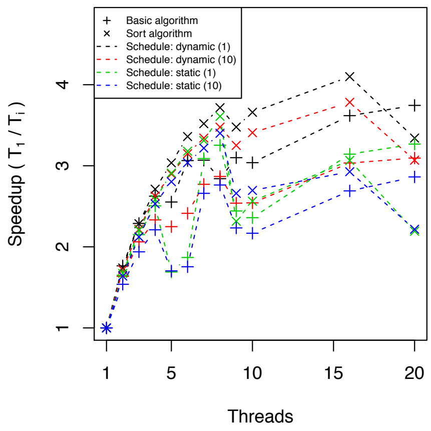

(b) FLUTE, randomized population.

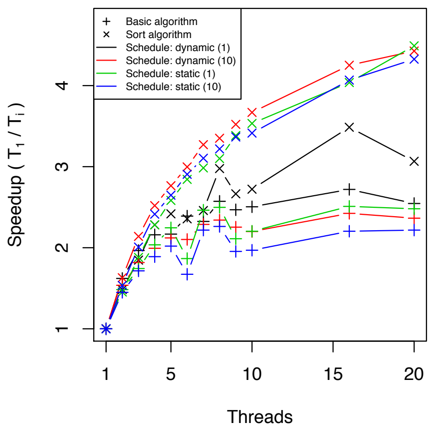

(c) FRED, sorted population.

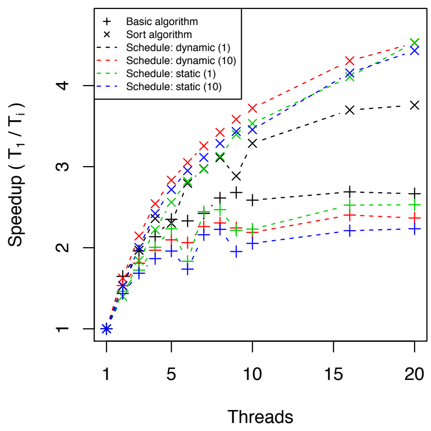

(d) FRED, randomized population.

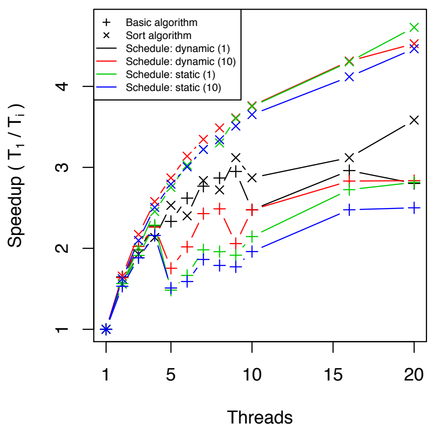

(e) SID, sorted population.

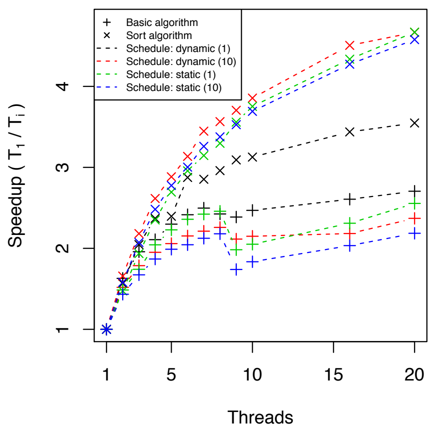

(f) SID, randomized population.

Figure S4: **Speedup according to thread number and scheduling for Brooklyn simulations on CPU type 1** Timing are shown for the basic and sort algorithm with dynamic and static parallel scheduling using workload chunk size of 1 and 10 clusters.

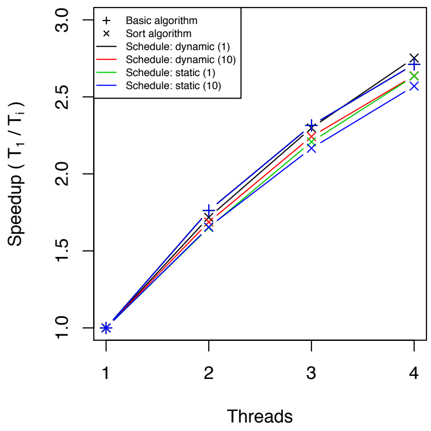

(a) FLUTE, sorted population.

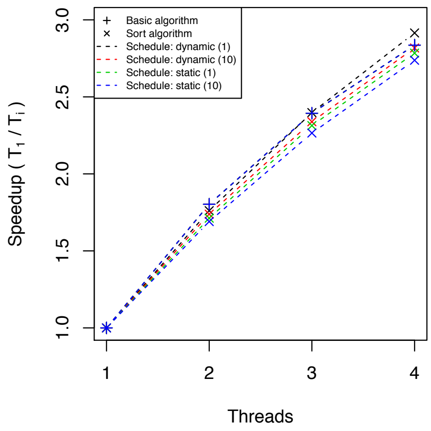

(b) FLUTE, randomized population.

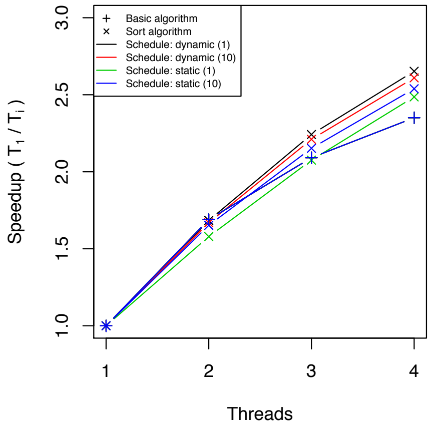

(c) FRED, sorted population.

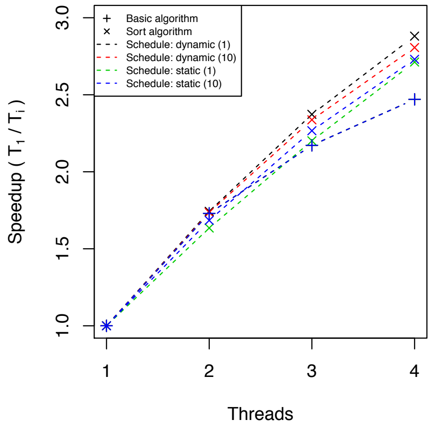

(d) FRED, randomized population.

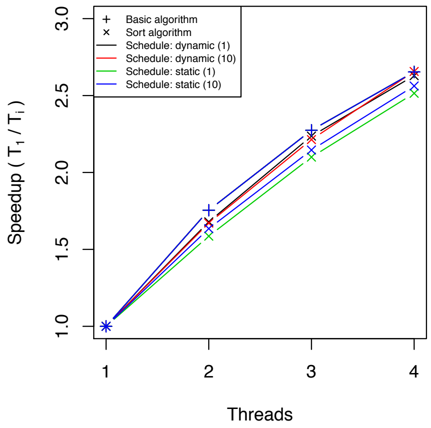

(e) SID, sorted population.

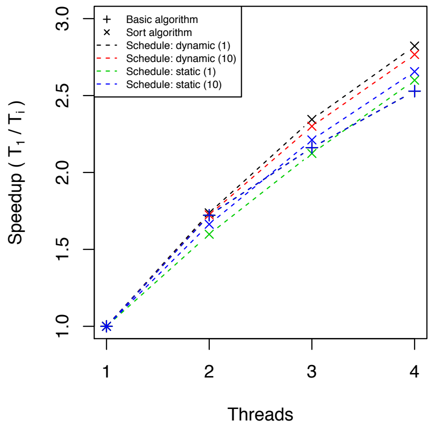

(f) SID, randomized population.

Figure S5: **Run times according to thread number and scheduling for Brooklyn simulations on CPU type 2** Timing are shown for the basic and sort algorithm with dynamic scheduling using workload chunk size of 1.
