# Supplementary material for: Optimizing agent-based transmission models for infectious diseases
Source: BMC Bioinformatics. 2015 Jun 2;16(1):183. doi: 10.1186/s12859-015-0612-2 (PMC4450454; doi:10.1186/s12859-015-0612-2)
Supplement: Additional file 5 — Model exploration and validation. Benchmarks to assess the impact of model complexity on model performance. [file 12859_2015_612_MOESM5_ESM.pdf]

Additional file 5 : Model exploration and validation.

The complexity of the transmission model presented in the main text is limited and person autonomy is constraint. To demonstrate that our findings are also useful for more complex models, we explored four aspects of transmission models: cluster size, dynamic clusters, number of person attributes, person autonomy. Information on the original population dynamics and implementations can be found in the main text.

Cluster size

Instead of using population data from RTI International, we created a virtual population of 1 million people with ages between 0-94 and assigned them randomly to predefined home and day district clusters. Households, schools and workplaces were not included in this population structure. Each district cluster had the same size and we repeated the procedure for size 20, 500 and 10000. These populations were used in a randomized and sorted order to simulate epidemics with seeding rate $1e^{-4}$ and attack rate ± 0.65, analogously to the main text. To obtain similar attack rates with different cluster sizes, we needed to adjust the transmission rates: E.g., to obtain on average 2 secondary cases, the transmission rate for cluster size 20 should be around 0.1 whereas this rate should be around 0.0002 for cluster size 10000. Also the network dynamics differ with the altered cluster sizes. Figure S1 illustrates the attack rate distributions from 10 simulations with each cluster size and population structure.

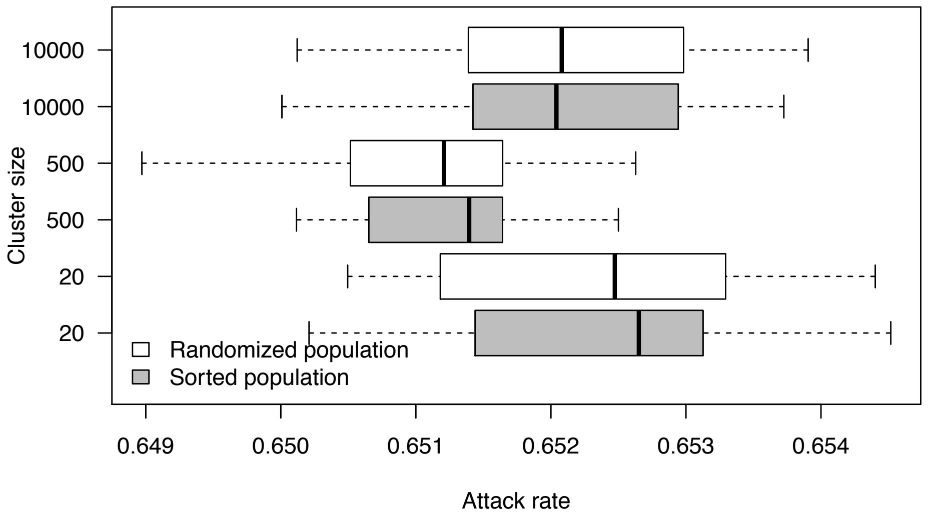

Figure S1: **Attack rate according to cluster size and population structure.** Results from 10 simulations. Box = upper and lower quartile, wiskers = minimum and maximum excluding outliers.

Table S1 presents the mean run time for simulations with attack rate ± 0.65 given the cluster size and population structure. We observed that cluster size had a large impact on run time because it affects the size of the 2 nested loops in the disease

transmission processing (for each infected => for each susceptible). The run times for simulations with clusters size 20 are dominated by model initialization (±5s). Sorting the population was beneficial for model performance irrespective of cluster size or model design and the benefit increased with incremented cluster size. The differences in run time regarding model design (FLUTE, FRED and SID) did scale with cluster size and the sort algorithm performed better than the basic algorithm.

Table S1: **Run time according to cluster size and population structure.** Mean times are presented from 10 independent realizations.

Cluster size	Population	FLUTE	FLUTE [sort]	FRED	FRED [sort]	SID	SID [sort]
20	Randomized	8	8	7	7	7	6
20	Sorted	6	6	6	6	6	5
500	Randomized	23	19	22	15	21	15
500	Sorted	20	16	19	14	18	13
10000	Randomized	519	287	414	200	432	205
10000	Sorted	373	229	307	176	308	166

Dynamic clusters

To estimate the effect of dynamic clusters on model performance, we adapted the original implementation so that membership to a social-contact cluster can change over time. All simulations started with the original RTI International population for Nassau and each time step 200 000 people (15% of the population) were assigned to a new day district. We randomly selected two members from two random day clusters and switched their day district. To focus this analysis, cluster sizes remained constant and only day districts could change. Within FLUTE, it is not possible to separate large district clusters from subclusters like schools or workplaces and therefore we implemented this extension only for FRED and SID. Dynamic clusters improved disease transmission so we needed to adjust transmission rates to obtain similar attack rates. Figure S4 at the end of this document illustrates the attack rate distribution for each implementation.

Figure S2 presents the run time for simulations with the original and dynamic clusters using a sorted or randomized population. The run time increased with the introduction of dynamic clusters although the ranking of the models on run time did not change (from high to low: FRED, SID, FRED sort, SID sort). The impact of the dynamic clusters was similar for sorted and randomized populations.

Person attributes

People in the original implementation had 11 attributes: id, age, household, home district, day cluster, day district, disease counter and four health state booleans (susceptible? — infected? — infectious? — recovered?). To estimate model performance with more elaborated person implementations, we added 30 data members to each person. For FLUTE and FRED, these values were added to the *Person* class as an array and initialized with random doubles in the constructor. For SID , we added 30 vectors to the *Population* class and each vector was extended with a random double when a person was added to the population. The new person attributes were not used in the program. The transmission dynamics or attack rate did not change compared with the original implementation (Figure S4).

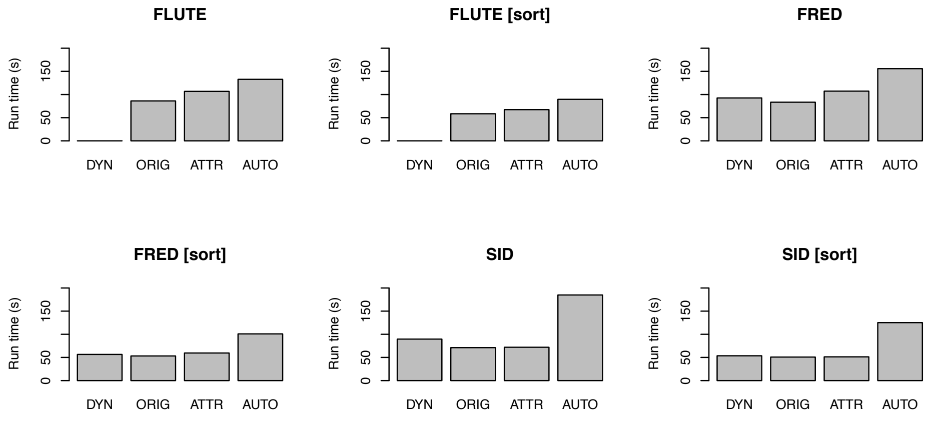

(a) Sorted population.

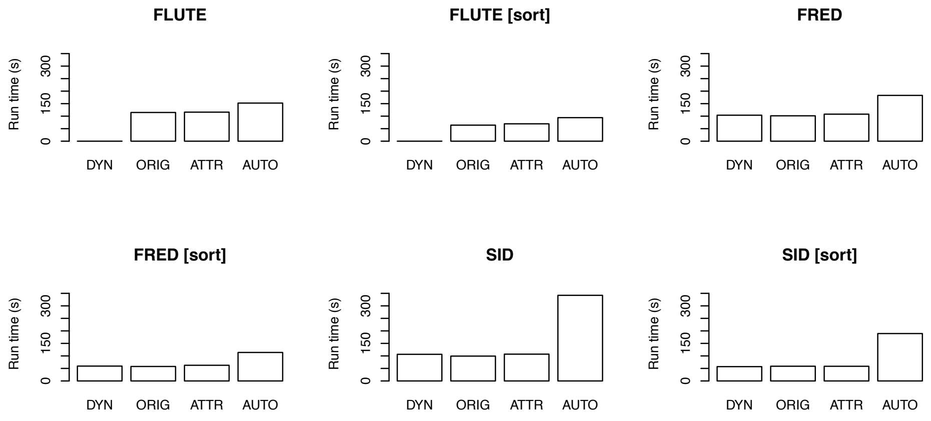

(b) Randomized population.

Figure S2: **Run time according to model implementation.** Mean run times from 10 simulations with AR of ± 0.64. DYN: dynamic clusters, ORIG: original implementation, FEAT: extended person features, AUTO: extended person autonomy.

Figure S2 presents the mean run time for simulations with and without the extra person attributes. The run time for FLUTE and FRED increased using the extended person features but the run time for SID remained constant. Interestingly, the timings for FLUTE and FRED did not change with the randomized population. We analyzed the effect of the extra person attributes for FLUTE and FRED with the profiling tool PerfExpert and observed that the amount of low level cache misses using the sorted population became similar to the amount of cache misses when the randomized population was used. We did not observe an increase in cache misses due to the extra person attributes with the randomized population. These results confirmed the targeted data management strategy using struct-of-array vs array-of-structs as stated in the main text.

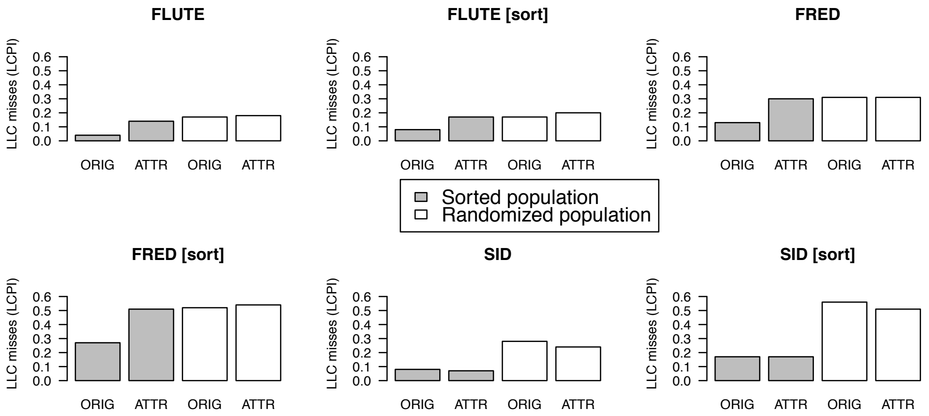

Figure S3: **Last level cache (LLC) misses with the original and extended person feature implementations with respect to population structure.** ORIG: original implementation, FEAT: extended person features, LCPI: local cycles per instruction

Person autonomy

To explore increased person autonomy, we used the random values that were stored in the extra person attributes (see previous section). We did not include an extra random number engine to focus in this extension on person autonomy rather than on calculating random numbers. We extended the decision process whether an infectious person is able to transmit the disease. This might be equivalent for choices like: to stay home, taking precautions,... To be able to compare model performance, we needed to increase the workload and branch instructions without changing the disease dynamics and simulation outcome. Therefore we implemented the following procedure with disease_counter {0-6} and attribute[i] {0-10}:

Function to check whether person x is able to transmit a disease:
1. **Set** probability = 0
2. **For** all extra attributes i in Person
3.   **Set** probability = probability + 1 / (disease_counter * attribute[i] + 1)
4.   **If** probability > 1
5.   **Set** probability to 0
6. **If** probablity < 0
7.   **Return** false
8. **Else**
9. **Return** health_state_is_infectious?

The original function contained only the last statement. The extended function did not alter the transmission dynamics since the *probability* could never be < 0 and the default random number generator was not used. The feature values and math function (line 3) were chosen so that program errors could not occur. For FLUTE and FRED , we introduced this extension in *Person* with a loop over the array containing the extra person attributes. Modifying SID required a lot more work since each attribute vector needed to be consulted separately.

All run times increased with the extended person autonomy, independent of the population structure, though we observed most difference in the timings with SID (Figure S2). The latter seemed not suited to handle many person attributes. Together with the increased workload, these timings demonstrate the disadvantage of the SID implementation.

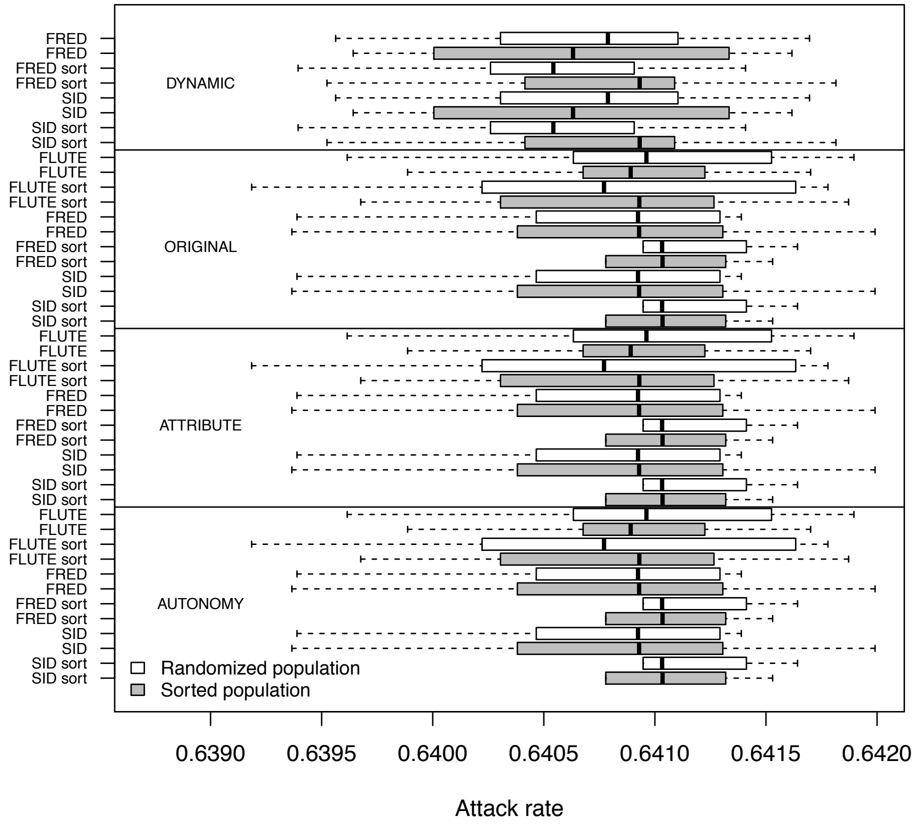

Figure S4: **Attack rate distribution according to model implementation and population structure.** Results from 10 simulations. Box = upper and lower quartile, wiskers = minimum and maximum excluding outliers.
